# Isn't it ironic? Confessions of a pregnant, iron deficient hematologist

**DOI:** 10.1002/hem3.70450

**Published:** 2026-08-10

**Authors:** Lauren E. Merz

**Affiliations:** ^1^ Division of Hematology/Oncology, Department of Medicine University of Michigan Ann Arbor MI USA

It is well documented that iron deficiency (with or without anemia) has an atrociously high prevalence among pregnant people (up to 80%), causes myriad symptoms that impact quality of life and maternal–fetal outcomes, and treatment simply involves replenishing iron stores with intravenous (IV) or oral (PO) iron supplementation.^1^, ^2^, ^3^ It should not be difficult for anyone to obtain testing and treatment for iron deficiency during pregnancy. And yet, here I am: a hematologist specializing in women's health, currently 33 weeks into my first pregnancy, and struggling to obtain iron testing and treatment. The evidence around iron deficiency in pregnancy is clear—why does it remain so difficult to test and treat?

At my very first obstetrics visit around 9 weeks of gestation, I asked my obstetrician if we could add a serum ferritin to the routine blood work planned after the visit. After a slight pause, I rushed to remind her that this was a hazard of caring for a hematologist. Likely as a professional courtesy, she agreed to my request. However, when attempting to sign the order, a warning flashed on the screen indicating that a single serum ferritin test linked to the billing code of an initial prenatal care encounter would not be covered by my insurance plan and could cost hundreds of dollars. Flustered, we both agreed to skip testing for now and continue taking prenatal vitamins that included the recommended 28 mg of iron daily. As we parted, she stated, “Don't worry—if your hemoglobin is low, we can definitely check the ferritin!”

My excellent obstetrician was quoting from the 2008 American College of Obstetricians and Gynecology (ACOG) Practice Bulletin, which indeed recommends screening for anemia in the first trimester with a complete blood count, but only recommends iron testing if anemia is detected.^4^ Additionally, 2024 guidance from the US Preventive Services Task Force (USPSTF) concluded that the current evidence is insufficient to assess the balance of benefits and harms of screening for iron deficiency in pregnant people to prevent adverse maternal and infant health outcomes.^5^ However, I was not reassured by this approach nor these guidelines. One study in pregnant women presenting for their first prenatal visit between 8 and 10 weeks of gestation with hemoglobin of 12 g/dL or higher (non‐anemic) found that over a third were already iron deficient.^6^ In recognition of this reality, both the European Hematology Association (EHA) and the International Federation on of Gynecology Obstetrics (FIGO) recommend regular assessment of both hemoglobin and iron studies as early as possible in pregnancy.^2^, ^7^

A few weeks later, my savvy primary care physician managed to order a serum ferritin test with an insurance‐approved billing code. My ferritin was 34 ng/mL. While the World Health Organization (WHO) guidelines have recommended using a cutoff of ferritin < 15 ng/mL to diagnose iron deficiency, this value has been questioned in the past few years.^6^ In fact, the soon‐to‐be‐released American Society of Hematology (ASH) guidelines for diagnosis of iron deficiency suggest that a ferritin threshold of <30 ng/mL should be used for pregnant patients and suggest that a serum ferritin of <50 ng/mL is appropriate for pregnant individuals with anemia and/or other iron deficiency risk factors and/or symptoms associated with iron deficiency. I decided that the cloud of fatigue and brain fog that followed me around might be due to iron deficiency, so I started taking an additional iron supplement by mouth as often as I could tolerate the associated wave of nausea in addition to my daily prenatal vitamin with iron. This is a major concern in pregnant patients—common side effects of PO iron include constipation, dyspepsia, and nausea, which overlap with and worsen common pregnancy symptoms, significantly limiting tolerability.^3^

As I entered the third trimester, I once again requested ferritin testing per EHA and FIGO guidelines.^2^, ^7^ I was also compelled by a recent US‐based study which found that second‐ and third‐trimester screening at a threshold of ≤30 ng/mL for ferritin is cost‐effective and improves quality‐adjusted life expectancy for mothers, even without accounting for any fetal benefit.^8^ After a pause, my obstetrician said, “Okay… but you were normal in the first trimester.” I refrained from replying that a pregnant patient's iron requirement of >1000 mg throughout gestation was predominantly concentrated in the second and third trimester, and over 80% of pregnant individuals in high‐resource settings are iron deficient by the third trimester.^1^ To my vindication (and dismay), my ferritin returned at 11 ng/mL despite PO iron intake at near‐maximal tolerated levels over the last few months.

My obstetrician offered me IV iron, and we agreed on a single dose of 1000 mg of iron dextran over 60 min in a single visit to maximize iron repletion in the shortest time and minimize my time at the infusion center. However, we soon learned that the infusion center had recently adopted a policy of administering iron sucrose only to pregnant patients, at 200–300 mg over 90 min given over 4+ sessions and no more often than once weekly, due to concerns about recent excess “reactions” to single‐dose iron dextran. On chatting with my infusion nurse during the first session, it appeared that most of these concerning episodes were likely Fishbane reactions, which indeed impact 1%–3% of iron infusions.^8^ Common symptoms such as flushing, back or chest pressure, mild dyspnea, mild tachycardia, or mild hypotension are distressing and can mimic anaphylaxis, but are distinguished by the absence of urticaria, bronchospasm, or vascular collapse.^3^ Importantly, symptoms resolve within 5–10 min of stopping the infusion, do not require additional intervention, and usually permit resumption of the infusion.^8^ Despite regularly administering IV iron infusions, she had never heard of Fishbane reactions, let alone received formal training in their identification and appropriate treatment. This is a common training gap and a disservice to our patients and infusion providers.

My frustration with this policy change was compounded when I considered that a US‐based study recently found that IV iron sucrose supplementation incurred significantly higher total costs ($163,500) than IV iron dextran ($157,100). In fact, most of the excess cost from iron sucrose is from the personal and economic burden of wages and time lost commuting to and from and undergoing five iron infusions instead of a single dose.^9^ I was feeling this intimately as nearly a half‐day slipped away between commuting, check‐in, vitals, IV placement, 90‐min infusion, and the 30‐min observation period. I have the privilege of reliable transportation, no childcare considerations, and a flexible job. How can we accept asking patients to manage this drain on their time and resources 5× over, without any significant reduction in serious adverse events compared to single dosing?

Furthermore, infusion chairs at my institution (as many other places) are in short supply, which means that my final dose of IV iron will be administered over 2 months after my initial diagnosis in the early third trimester and a mere 2 weeks before my due date. In addition to a significant impact on quality of life, iron deficiency in pregnancy has significant impacts on maternal outcomes including higher rates of preterm birth, cesarean delivery, preeclampsia, postpartum depression, severe postpartum hemorrhage, need for blood transfusion, and even maternal death.^10^, ^11^ Furthermore, iron deficiency in pregnancy is associated with inferior fetal outcomes including low birth weight, increased risk of neurocognitive issues, and reduced social engagement and motor behaviors.^12^, ^13^ Rapid identification and correction of iron deficiency are critical for maternal quality of life and for optimizing maternal and fetal outcomes.

I write this as I sit in the infusion center receiving my second dose of IV iron. I am grateful to my thoughtful and skilled obstetricians who were willing to collaborate with me and recognize where the current US guidelines fall short. My story is shared by many pregnant people, most of whom do not have the luxury of being a hematologist specializing in women's health to help break through numerous barriers to obtain necessary iron testing and treatment. Pregnancy is hard enough. Integrating actionable workflows for timely iron screening and rapid, cost‐effective treatment of iron deficiency is an easy way to smooth the road.

## AUTHOR CONTRIBUTIONS

**Lauren E. Merz**: Conceptualization; writing original draft; writing review editing.

## CONFLICT OF INTEREST STATEMENT

The author declares no conflicts of interest.

## FUNDING

This research received no funding.

## Data Availability

Data sharing not applicable to this article as no datasets were generated or analyzed during the current study.
